# Indents

**Published:** 1915-02

**Authors:** 


### INDENTS.

**☞ Brief Articles of Technical or Scientific Value will receive notice and comment. Contributions invited.**

*Hand Lotions and Chapped Hands.* Hand lotions are generally of two types: (1) glycerin solutions of the glycerin, rose water and benzoin kind, and (2) gelatinous liquids made with tragacanth, quince seed, or some other water-soluble colloid. The glycerin lotions work well with some skins and are the most satisfactory to an occasional individual, but as a rule they are not the best, first, because the glycerin tends to make drier a skin already too dry, and, second, because these lotions have little or no detergent effect and do not clean the skin.

The tragacanth and quince seed lotions are better, and of these the tragacanth lotions are certainly as good as any other and are very cheap and easy to make. A formula for such a lotion given by Pusey is as follows:

| | |
|---|---|
| Tragacanth .................. | gr. 80 |
| Glycerin ..................... | ℥ ss |
| Boric acid .................... | ℥ ss |
| Water ....................q. s. | Oi |
| Oil of bergamot................ | ♏ iv |
| Oil of lavender................ | ♏ ii |
| Oil of rose.................... | ♏ i |

The oils mentioned being added as a perfume might be omitted, one or all of them, according to the wish of the prescriber.

The boric acid, glycerin and waters are first mixed, the tragacanth added and the mixture agitated until the tragacanth is dissolved. This makes a rather thick mucilage; it can be changed to any consistency desired by slight increase or decrease in the amount of tragacanth.

A lotion like this has a considerable detergent effect; it is a tolerable substitute for soap, and if it is freely rubbed over the hands and wiped off, either with or without the use of water, cleans the skin of all but the most tenacious dirt. It, of course, can not be effectually used as a complete substitute for soap. Such a lotion has the advantage over soap that it not only is not irritating to sensitive skin but also is bland and soothing. It thus tends to prevent and eventually to cure chapping of the hands.

The chief reason for chapping of the hands is the lack of fat in the skin in cold weather. Fat production in the skin is at a minimum in cold weather, because of the diminished sebaceous and sweat secretion. This and the dry air of winter make the skin dry and vulnerable at the very time when the cold air is itself irritating. This combination leads readily to chapping, if the hands must be exposed much to soap and water, and particularly if the irritation of antiseptics is added, as in the case of physicians and nurses.

The first thing to do to prevent or overcome the condition is to supply, by greasing the skin occasionally, the lacking fat in the skin. Almost any bland fat or semisolid hydrocarbon will do for this purpose, but nothing is better than a well-made cold cream. The next and the more difficult thing to do is to avoid soap and water—especially soap—as much as possible; and it is here that hand lotions serve a very useful purpose.—*Journal A. M. A.*

*Dental Commandments.* The Dental Department of McGill University in Montreal, Canada, has formulated the following code for distribution among its soldiers for the European war:

1—Lord Roberts said, "We live by our teeth"; therefore keep your teeth in the best possible condition.

2—The mouth is nature's great incubator and center of infection; therefore keep it clean.

3—Pus is always exuding from about the roots of diseased gums and teeth; therefore have all such roots extracted.

4—Small cavities soon become dangerously large; therefore have all decayed teeth filled.

5—Digestion begins in the mouth; to avoid indigestion, keep the mouth in condition to do its complete work.

6—Starchy foods require saliva for their full digestion. This must be supplied whilst the food is in the mouth; therefore chew the food until it is completely insalivated.

7—Clean teeth do not decay; therefore they should be brushed at regular intervals to keep them clean.

8—A large brush can not be properly used in the mouth, a stiff brush will injure the gums; therefore use a small brush which is not too stiff.

9—Food particles adhering to the teeth form the nourishment for bacteria; therefore brush the teeth after each meal and before retiring.

10—Any good dentifrice will assist in cleansing the teeth, but lots of water is necessary in every case.—*Dominion Journal.*

*Medical Prisoners Exchanged.* The several warring nations are exchanging captured prisoners of war. This seems one of the sensible things that has come to front in this insane butchery of the best blood of the warring countries. It seems necessary that some such exchange be made that sufficient surgeons and physicians may be had to take proper care of the horrible results of the war.

*Novocain and the War.* One of the drugs much used in dentistry that we are dependent on Germany for is Novocain. It is to be hoped that arrangements will soon be made that will secure shipments of this drug through the lines. The company making it in England closed its factory, as it was almost entirely owned by Germans, but the English Government has reopened all such factories and will manufacture its own drugs. The Government has subsidized a new English factory for the manufacture of Analine dies. It would seem to be a strategic move on the part of American drug manufacturers that they make such business arrangements as may be practicable with German manufacturers to produce these valuable drugs on our own territory. In England a disinfectant called Densol has just appeared to take the place of Lysol. We have such in this country, but none have yet appeared that is an entirely satisfactory substitute for the German Lysol.

*Care of the Teeth in the Armies.* The English Secretary of War stated in the House of Commons, recently, that they had found diseases of the soldiers' teeth more prevalent than any other disease and that steps were being taken to have dentists appointed to look after them, and the general officers are empowered to spend the money necessary to fit any soldier for active service. A number of private practitioners are volunteering their services and several institutions have agreed to do whatever they can to care for the soldiers that can be reached by them. Dental surgeons are being appointed to all the larger military stations at home, who will devote all their time to the care of the teeth. At the front, it has been found necessary to do a great deal of extracting which has necessitated the insertion of dentures. To accomplish it has been found necessary to call in a large number of dental mechanics to take over this part of the work.

*Proportions in Which Teeth are Extracted.* Dr. A. H. Lemke has an interesting report in the *Bur* for January of the relative numbers of teeth extracted by him during the past six years. He kept a careful record of every permanent tooth extracted by him located on a chart as to its position in the jaws. He finds that he has extracted in the six years 923 teeth, 516 from the upper jaw and 407 from the lower jaw. Of the 516 upper teeth 246 were molars, 129 from the right maxilla and 117 from the left maxilla, 104 lower right molars and 148 lower left molars. 270 uppers included incisors, cuspids and bicuspids; 155 lower incisors, cuspids and bicuspids. The upper front teeth show almost double loss over the corresponding lower teeth. The lower left first molar was oftenest lost, next the upper right molar, about 60 per thousand. The tooth least often extracted was the lower left central, about 10 per thousand. Next to the molars the upper bicuspids are oftenest lost—the first bicuspid oftenest. This is an interesting study and bears out facts already brought by Black and others.

*The Doctors' Title for German Dentists.* German dentists who are not fighting the Allies, are having a hot time with the German physicians, who are violently opposed to the effort being made to allow the Universities to confer on dental gratuates the D. D. S. degree. The physicians claim that dentists must be educated as M. D. before they can be called doctors. The physicians say the dentists have no more right to a special doctorate degree than the ophthalmologist, or the surgeon. It's a pretty scrap and the probabilities are the physicians, being more numerous, will keep the Zahnarzts from changing their title.

*Alcohol as a Germicide.* According to the statement of the *Medical Wochenschrift*, seventy per cent. alcohol is a more satisfactory germicide than 100 per cent., or absolute, alcohol.

*Forsythe Infirmary.* At the dedication of this institution its founder said, "It is my wish that this building shall be as a home to the children, beautiful and cheerful; a protector of their health, and a refuge from pain. By making them healthier and happier I hope it may make them grow to be better citizens. If this is accomplished, as I believe it must be with the cordial co-operation of the dental profession, I shall feel that our gift has been well bestowed." This is certainly a lofty sentiment, and we predict that in years to come this will be one of the recognized epoch-making events in the history of preventive medicine.

*American Dental Hospital in France.* Americans in France are making an effort to raise $500,000 to carry on the work of a thoroughly equipped hospital for the treatment of wounded soldiers. $200,000 has already been contributed by Americans in Paris and London, and subscriptions in this country will be forwarded by J. P. Morgan & Co., the New York bankers. A most interesting and much-praised department of the work is the Dental Department of the hospital which is directly under the charge of Drs. Wm. S. Davenport and George B. Hays, both American dentists practicing in Paris. Any one desiring to contribute will send their money to J. P. Morgan & Co., New York, who will forward it at once. This is certainly a worthy cause and we may be assured that the dental work could not be placed in better hands.

*Results of Implantation.* I have found that when metal was retained in the jaw bone, the bone tissue did not take kindly to it: that roots denuded of peridental membrane were exfoliated; that autogenous and homogenous tooth roots with peridental membrane upon them were retained; that the alien tooth roots not nursed upon the blood of the individual into whom they are transplanted are lost (except in one instance, where the sheep tooth root was retained, but only mechanically); that alien grafts (tooth roots), when nursed upon the blood of the individual into whom they are transplanted, are retained; that powdered bone incorporated is vaseline, when introduced into tissue, invites calcareous deposits and the formation of new bone; and, best of all, that the possibility of transplanting alien tissue is no longer in doubt.—H. J. Kauffer, *Items of Interest.*

*First Aid to Those in Need of Artificial Respiration.* The Bureau of Mines has recently published ("Technical Paper 77") the report of the Committee on Resuscitation from Mine-Gases, the members of this Committee being W. B. Cannon, George W. Crile, Joseph Erlanger, Yandell Henderson, and S. T. Meltzer. One of the results of these investigations is the recommendation of the Bureau of Mines that whenever, for whatever reason, artificial respiration is instituted, the following methods be adopted:

In gassing (being overcome by gases), remove the victim at once from the noxious atmosphere. Carry him quickly out into the fresh air and instantly institute manual artificial respiration. Do not stop to loosen any clothing, for every moment of delay diminishes the chance of recovery.

In case of electric shock, break the electric current instantly. Free the patient from the current with a single quick motion, using any dry nonconductor (clothing, rope, glass, board) to move the patient or the charged wire. Beware of using for the purpose any metal or even moist material. Meantime have every effort made to shut off the electric current.

Attend instantly to the victim's breathing. If the victim is not breathing, manual artificial respiration must be resorted to at once.

If the patient is breathing slowly and regularly, do not employ artificial respiration, but let nature restore breathing unaided.

In gas-poisoning, administer oxygen, if available. Also employ manual artificial respiration. The oxygen is supplied through a breathing-bag from a cylinder. The latter, for this purpose, has a reducing valve, and is supplied with connecting tubes and face-mask, and an inspiratory and an expiratory valve, the latter communicating directly with the atmosphere.

No mechanical breathing device should be used for resuscitating, except it be one operated by hand and having no suction effect on the lungs.

Employ the Schaefer, or prone, pressure method of artificial respiration. Begin at once. A moment's delay is serious. Proceed as directed in ''Miners' Circulars'' Nos. 5 and 8 of the Bureau of Mines.

Patiently continue, without interruption, the artificial respiration, if necessary, for two hours or longer, until natural breathing is restored. If natural breathing stops after having been restored, again institute artificial respiration.

Do not give the patient any liquid by mouth until he is fully conscious.

Place him where there is fresh air, but keep his body warm.

Send for the nearest doctor as soon as the accident is discovered.

The Committee of the Bureau of Mines referred to above also discusses at considerable length, in ''Technical Paper 77,'' the four mechanical devices (pulmotor, the Brat apparatus, the lungmotor, and the salvator) that are now on the market and in quite common use for instituting artificial respiration. A careful analysis is made of the literature upon the subject of devices of this kind, and the final conclusion of the committee is that on the whole it disapproves of them, or at least of the pulmotor and the Brat apparatus, which two it has examined. The committee declare that these instruments are objectionable, because repeated suction of the air from the lungs is not physiological and, if continued, is likely to result in injury to the lungs and to inadequate inflation. It also disapproves of the pulmotor, because the automatic mechanism is so easily disturbed as to be very liable to fail at critical moments.

The committee, however, recommends the use of an apparatus devised by one of its members, Dr. S. T. Meltzer.

One of the objections to the pulmotor and similar apparatus, namely, the fact that it permits of entrance of air into the stomach, is guarded against by the use of the Meltzer apparatus. In addition, a weight is to be placed upon the patient's abdomen, in order further to restrict the entrance of air into the alimentary canal. It is declared that this apparatus is free from sucking action during expiration, is light, simple, inexpensive, is readily understood by laymen, and can be used to deliver pure oxygen. This is an important consideration in the treatment of gas cases. —*Clinical Medicine.*

*Does Pyorrhea Cause Iritis?* W. M. Beaumont, writing in *The British Medical Journal,* September 26, 1914 (p. 525), quotes Lang's statistics, to the effect that 64 per cent. of the cases of iritis attributed to sepsis are caused by pyorrhea alveolaris. He is convinced that this disease is undoubtedly the most frequent cause of this form of iritis, although, as he declares, "it can not be too insistently brought to mind that the patient with pyorrhea may also be a sufferer from syphilis or gonorrhea." However, in a patient suffering from this form of eye disease, careful examination should be made of the mouth and, if it is diseased, appropriate treatment should be instituted. Doctor Beaumont says that drastic treatment is warranted in these cases, and, if necessary, all foci of disease should be removed by extraction of the offending teeth. As he puts it, "The cure of iritis is of paramount importance, and it is better to lose thirty-two teeth than one eye."

Now that it has been demonstrated that we have in emetine hydrochloride a real specific for pyorrhea, through the use of which we can not only cure the disease but usually save the teeth at the same time, it is apparent that this alkaloid should be given a thorough trial whenever the iritis is complicated with Riggs' disease. However, the possibility of syphilitic infection should always be kept in mind. Wassermann tests will usually settle the diagnosis.—*Clinical Medicine.*

*Investing a Cast Cusp Crown.* In casting the cusps of a gold crown the sprue can be inserted from the inside of the band to the under cusp surface of the wax. By inserting it here, when cast, the cusps will be free from a sprue mark and can be easily polished. A bur will cut off the sprue. In long crowns, with this method of attaching the sprue, the band will not be as near the bottom of the casting ring.—F. S. Dilger, in *Review.*

*Iodine Stains.* Should, through any accident, iodine stains get on the linen of the patient while in the chair, the application of hypo, or fixing solution used in developing, will cause prompt removal.—*Journal of the Allied Societies.*

*Good Soap.* One of the most gratifying things a dentist can have is a good soap, which after using many times a day, does not leave the hands in a dry, cracking condition. This is prepared by putting five pounds of U. S. P. Green's soap into a five-quart plaster pail, fill with water, boil for twenty minutes and let cool slowly. Do not add alcohol for clarifying, but let stand for a few days when the clear amber-colored solution can be syphoned off. This is the most satisfactory soap for our purposes that I know of.—A. G. Loomis, in *Dental Review.*

*Casting Cusps for Banded Crown.* In constructing banded crown with cast cusps good results can sometimes be obtained by fitting band and placing it in position on root and then placing wax in position obtaining bite and removing wax pattern and treating it like an inlay, casting and afterwards soldering or sweating it into position in the band.

The chief advantages are that the wax pattern is not distorted as is often the case in removing it "in situ" in a closely fitting band, also the thickness of gold in cusps can be more readily adjusted and the union between band and cusps assured.—A. G. Salisbury, in *Review*.

